# Natural history and long-term follow-up of incidental thyroid nodules on CT imaging

**DOI:** 10.1093/bjr/tqaf002

**Published:** 2025-01-21

**Authors:** Eda Lyuman, Claire McArthur

**Affiliations:** School of Medicine, University of Glasgow, Glasgow, G12 8QQ, United Kingdom; Department of Radiology, Glasgow Royal Infirmary, Glasgow, G4 0SF, United Kingdom

**Keywords:** thyroid nodules, thyroid cancer, CT scan, thyroid malignancy, follow-up, incidence

## Abstract

**Objectives:**

Incidental thyroid nodules (ITNs) are found in up to 25% of CT scans. Increased use of cross-sectional imaging has contributed to the increased incidence of thyroid cancer worldwide. ITNs pose a management dilemma since nodule malignancy rate is 5%-15% but most cancers are indolent and prognosis in differentiated thyroid cancer is excellent. Study aims are to determine prevalence of ITNs ≥1 cm on CT scans, evaluate reporting practices, assess for emergence of clinically evident thyroid cancer during 13-year follow-up and assess interim nodule growth and clinical outcomes in nodules that were further investigated.

**Methods:**

Direct image review of 1499 consecutive CT scans that included the thyroid, performed during January 2009 in a large NHS health board was performed. Clinical data up to January 2022 was analysed in 150 patients with at least 1 ITN ≥1 cm.

**Results:**

ITN prevalence was 11% with mean patient age 70 years and mean nodule diameter 17.5 mm. 30% of ITNs were mentioned in the CT report. During the follow-up period 11% proceeded to thyroid ultrasound, 5% fine needle aspiration, and 2% diagnostic hemithyroidectomy with no thyroid malignancy found. One hundred twenty patients (80%) were deceased by the study endpoint, none from thyroid malignancy. No patients presented with clinically evident thyroid malignancy during follow-up.

**Conclusions:**

None of 150 ITN cases developed clinically evident thyroid malignancy in a 13-year follow-up period with 80% of patients deceased by the study endpoint from non-thyroid causes.

**Advances in knowledge:**

This would suggest that ITNs detected on CT do not require further investigation unless malignant appearances or significant clinical concern for thyroid malignancy.

## Introduction

Thyroid nodules are extremely common. Incidental thyroid nodules (ITNs), defined as “a nodule not previously detected or suspected clinically but identified by an imaging study” are amongst the most frequent incidental findings encountered on imaging.^1^

Prevalence of ITNs on CT ranges from 5% to 25%.^2^ This depends on the population studied and on reporting practices of the radiologist.

On encountering an ITN on a CT scan, the dilemma arises of whether or not to pursue further investigations. The main concern is missing malignancy.^3^ However, while 7%-15% of thyroid nodules ultimately prove malignant, the majority of these cancers are indolent and of limited biological significance.^4^ Prognosis of differentiated thyroid cancer is excellent with cancers classified as T1/T2 and N0, M0 having an estimated disease specific survival of 99%.^5^

There has been an increase in incidence of thyroid cancer, evident worldwide^6^ over the past 40 years. In contrast, mortality rate has remained stable. Incidence of thyroid cancer at autopsy has not changed over the last 60-70 years, suggesting no true increase in prevalence. Therefore, it is presumed that increasing incidence is due to increased detection of subclinical cancer.^7^ Increased cross-sectional imaging use, with ever more advanced imaging techniques, has led to an increase in ITN detection.

A non-selective ultrasound and/or biopsy approach in ITN management can lead to overdiagnosis and psychological burden to the patients as well as increased cost to the healthcare system.

While CT and magnetic resonance imaging (MRI) are not recommended to be used in classifying ITNs as benign or malignant, they can demonstrate worrisome features such as extrathyroidal extension and associated lymphadenopathy in malignant cases.

**Figure 1.**
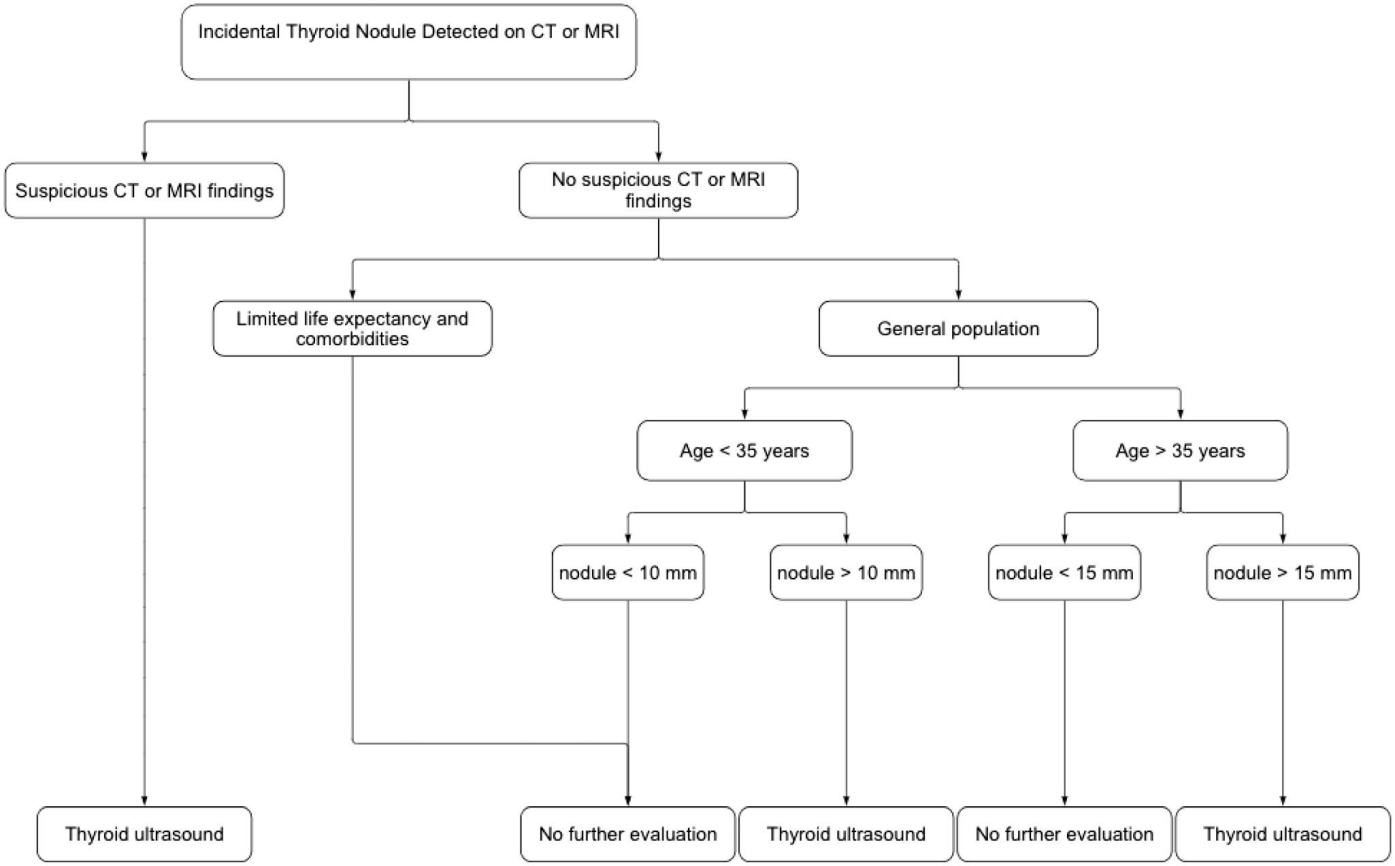
Flow chart showing evaluation for incidental thyroid nodules detected on CT or MRI adapted from Hoang JK et al. Managing Incidental Thyroid Nodules Detected on Imaging: White Paper of the ACR Incidental Thyroid Findings Committee. J Am Coll Radiol. 2015;12(2):143-50.

In 2015, The American College of Radiologists (ACR) Incidental Thyroid Findings Committee published a recommendation for thyroid nodules, which should undergo further evaluation, illustrated in Figure 1.^1^ This system takes into account suspicious findings on CT/MRI, for example, abnormal lymph nodes or invasion of surrounding tissues by the thyroid nodule, patient’s comorbidities and life expectancy, age of the patient and nodule size with ultrasound assessment recommended as shown in the figure. Mhuircheartaigh et al.^8^ concluded that these guidelines are effective at detecting thyroid cancers through ITN follow-up.

National Institute for Health and Care Excellence (NICE) guidelines on Thyroid Disease: Assessment and Management, 2019, recommend that “thyroid ultrasound of incidental findings should not be the default option because most incidental findings are not malignant and further investigation may cause harms in terms of the adverse effects of testing and patient anxiety.”^9^

British Thyroid Association (BTA) guidelines, 2014, advise clinical examination of these nodules: “In the majority of cases, no further assessment/investigation will be required. However, if there are suspicious findings on CT (extra thyroidal extension, tracheal invasion, associated suspicious lymphadenopathy), the patient belongs to a high-risk group or there is significant clinical concern, ultrasound assessment is recommended.”

Using the proposed size threshold in ACR guidelines would result in a significant proportion of ITNs requiring ultrasound assessment. On the other hand, BTA and NICE guidelines mean that either advanced malignancy must be present before ultrasound is recommended or that the reporting radiologist or requesting clinician is cognizant of any patient risk factors for thyroid cancer. The requesting clinician, often not a thyroid surgeon or endocrinologist may not feel confident determining the clinical likelihood of malignancy, particularly as many ITNs are impalpable. This reflects the need for a safe, cost-effective, evidence-based approach for evaluating ITNs.

The main aims of this study were to determine the prevalence of ITNs on CT imaging, evaluate the short and long-term outcomes in patients with ITNs ≥10 mm in diameter and appraise past reporting practices. Outcomes include rate of clinically evident thyroid cancer at 13-year follow-up, interim nodule growth and clinical outcomes in nodules that were further investigated during the follow-up period.

## Methods

Of an initial complete list of 3081 CT scans performed during the calendar month of January 2009, 1499 consecutive CT scans in adult patients within our Health Board during a 19-day period, where the thyroid gland was potentially included in the coverage, were examined on the Radiology Picture Archive and Communication System (PACS). These studies comprised thoracic CT, neck CT, cervical spine CT, thorax, abdomen ± pelvis CT, CT aortic arch, CT carotid angiogram, and CT pulmonary angiography (CTPA). Of these, 47 scans did not sufficiently cover the thyroid gland, 17 were not assessable due to poor image quality, 15 had no images available on PACS, and 5 were duplicate entries, ie, the same patient scanned more than once within that time period. CT scans were assessed by author 1, who was blinded at that point to the radiology report and patient clinical details. In any scans where the definitive presence of a thyroid nodule was equivocal, the images were also reviewed by blinded author 2 with 8 years of head and neck imaging experience.

**Figure 2.**
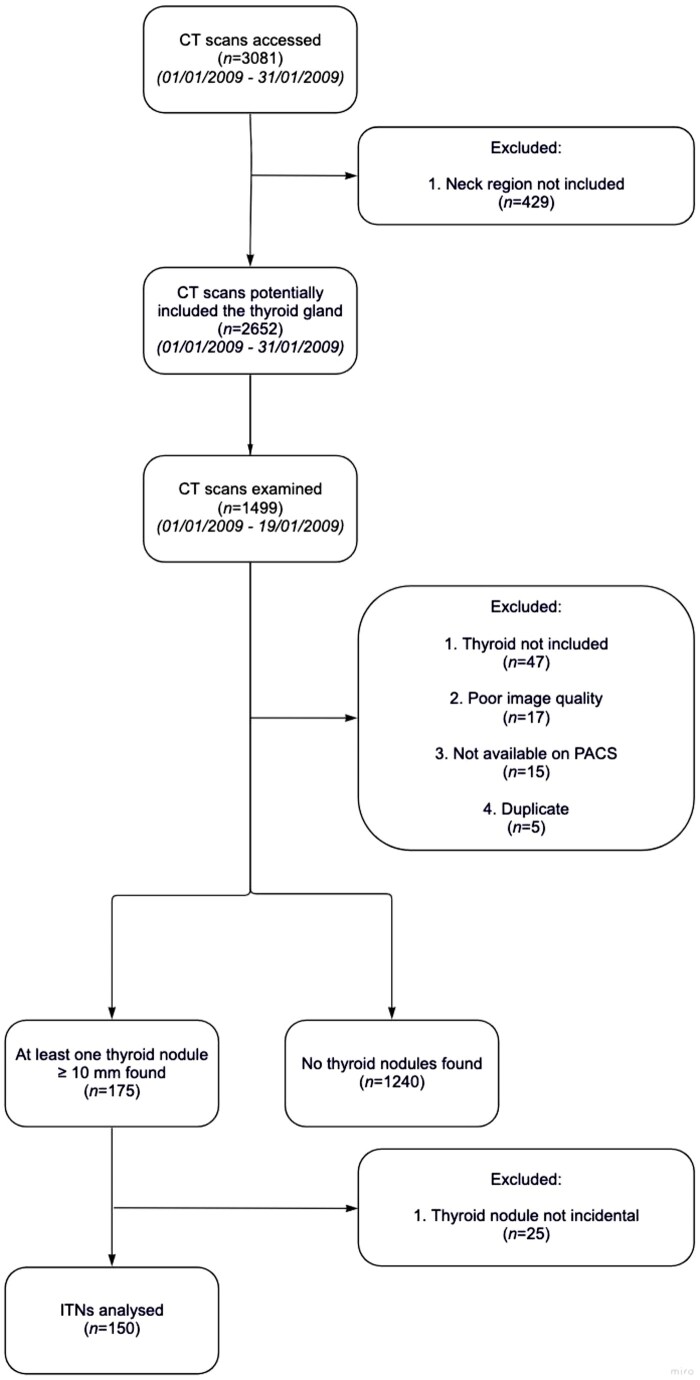
Flow chart summarizing study methods. ITN = incidental thyroid nodule; PACS = picture archive and communication system.

Of the remaining 1415 scans, at least 1 thyroid nodule ≥10 mm was detected in 175 patients (12%). Follow-up of 175 patients was carried out using electronic healthcare records. Twenty-five were excluded since the nodule was not truly incidental. Overall, 150 patients with ITN were included for further analysis. Methods are summarized in Figure 2.

The recorded parameters were sex, age at CT scan, presence of known risk factors for thyroid cancer, indication for CT scan, laterality and size of the nodule (longest diameter in mm), and presence and morphology of nodule calcification, whether small, coarse or rim (small being foci of approximately 1-3 mm, coarse referring to larger clumps or more globular calcifications and rim being linear calcification at any point at the nodule margin). If multinodular, the size of the largest nodule was recorded. We recorded whether the nodule was reported and if there was recommendation for further action in the CT report, whether there was clinic attendance, thyroid ultrasound (US)/fine needle aspiration (FNA)/thyroid surgery in the follow-up period with relevant results and health status (alive/deceased) with cause of death. Any further cross-sectional imaging that covered the thyroid in the follow-up period and if so, changes to the nodule size were also documented.

The follow-up period is defined as any time after the patient’s initial CT scan in January 2009 up to February 2022. Nodule growth rate on CT was calculated using the formula “growth rate = maximum diameter of nodule on follow-up CT (mm)/maximum diameter of nodule on initial CT (mm).”

### Statistical analysis

Results are described as percentage, mean, and standard deviation in the form of “mean (±standard deviation)” where applicable. Statistical analysis was performed with Minitab version 19.2020.2.0.10.

## Results

### Patient demographics

**Figure 3.**
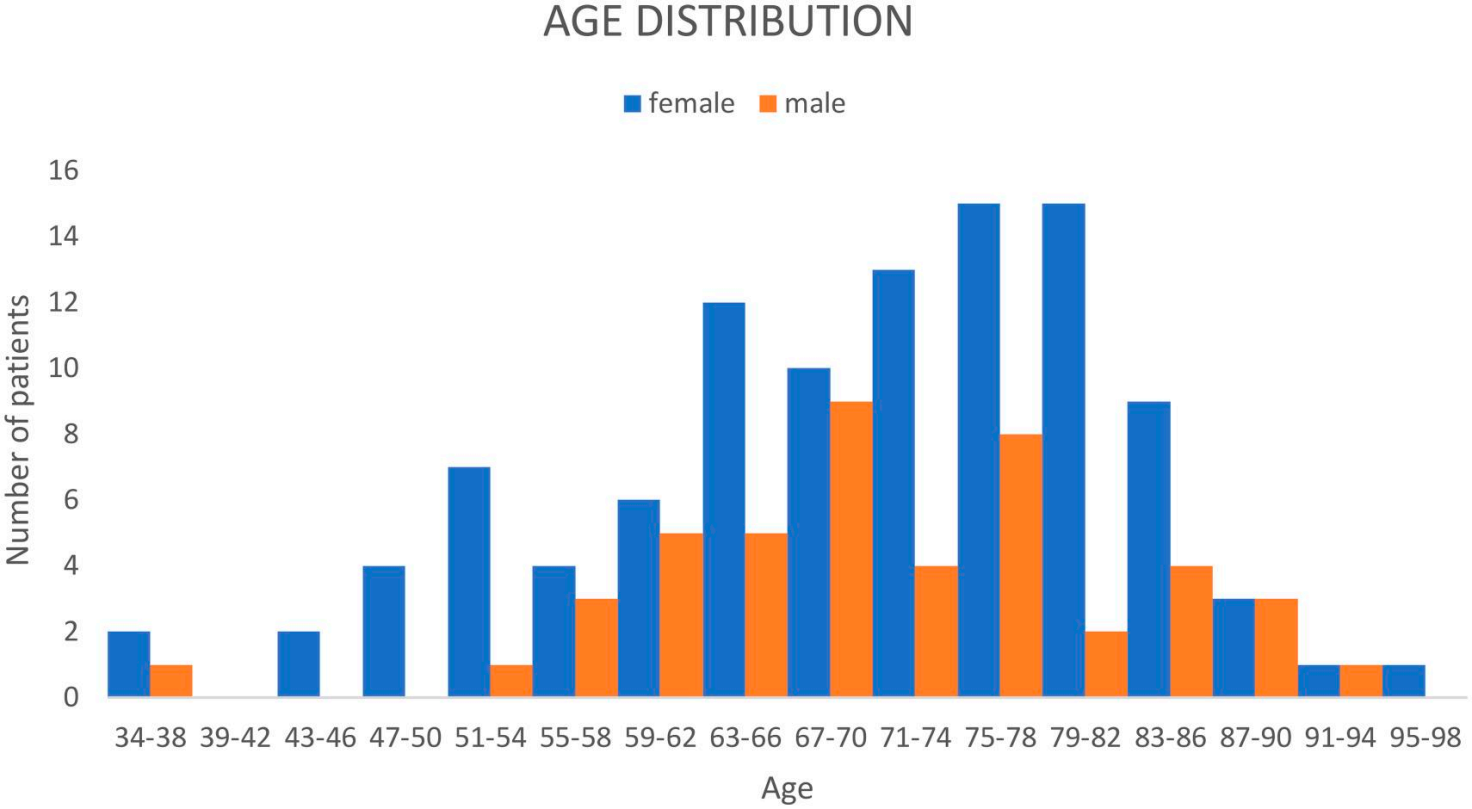
Histogram showing age distribution of the sample, stratified by sex.

Sixty-nine percent (*n* = 104) of patients were female. Mean age at the time of CT was 70 (±12) years. A histogram presenting the age distribution in this sample is seen as Figure 3. None of the patients were known to have any risk factors for thyroid cancer. Eighty percent (*n* = 120) of patients were deceased by the study endpoint.

**Figure 4.**
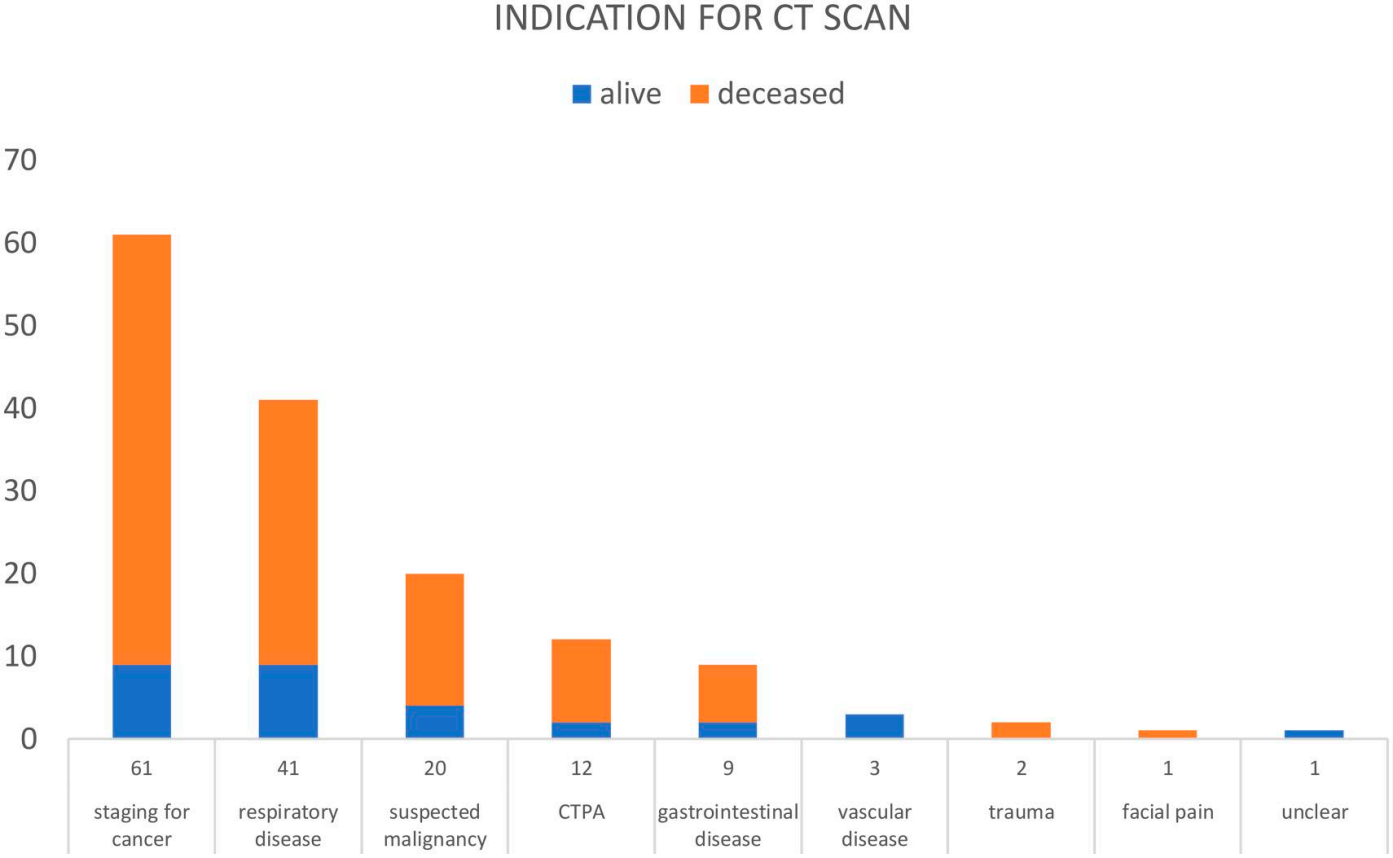
Bar chart showing the number of alive and deceased patients at the time of data collection in February 2022, stratified by the indication for CT scan performed in January 2009.

Figure 4 represents the proportion of alive and deceased patients at the study endpoint for each CT indication. The most common reason for CT scan was cancer staging (*n* = 61, 41%). For 120 deceased patients, cause of death was recorded when the death certificate was accessible via health records, shown in Table 1. There were no deaths attributable to thyroid cancer.

**Table 1.** Cause of death for deceased patients.

Cause of death	Number of patients	Percentage (%)
No data available	54	45
Cancer (non-thyroid)	37	31
Respiratory disease	14	12
Sepsis	5	4.2
Cardiovascular disease	5	4.2
Trauma	2	1.7
Liver disease	1	0.8
Renal failure	1	0.8
Neurodegenerative disease	1	0.8

### Nodule characteristics

ITN prevalence in the study sample was 11%. Mean size of the largest ITN was 17.5 mm (±9.3), range 10-53. The largest nodule was on the right lobe, left lobe and isthmus in 51%, 43%, and 5%, respectively. Forty-eight nodules (32%) were calcified, with small, coarse, and rim calcification in 13, 24, and 11 cases, respectively.

### Reporting practices

With reference to the age and size recommendations from ACR ITN committee, 63 patients (43%) in our sample were over the age of 35 with a thyroid nodule >15 mm and 2 patients (1.3%) were under the age of 35 with a thyroid nodule >10 mm.

**Figure 5.**
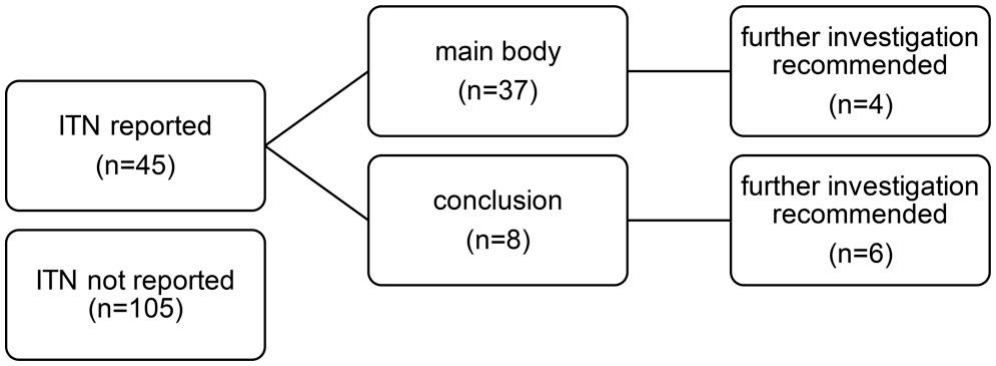
Reporting practices for incidental thyroid nodules.

Overall, 30% (*n* = 45) of the ITNs were reported in the CT report, 82% of these (*n* = 37) in the main body and 18% (*n* = 8) in the conclusion, Figure 5. Further investigation was recommended in 22% (*n* = 10) of the reported cases. The main recommended further investigation was a thyroid ultrasound.

### Long-term follow-up

Eleven percent (*n* = 5) of patients who had the ITN reported were seen in clinic for their thyroid nodule by either the original referrer, ear, nose, and throat (ENT), endocrine or general surgery clinician; 5.7% (*n* = 6) of patients who did not have their ITN reported in the CT scan were seen at a clinic regarding their thyroid nodule.

**Figure 6.**
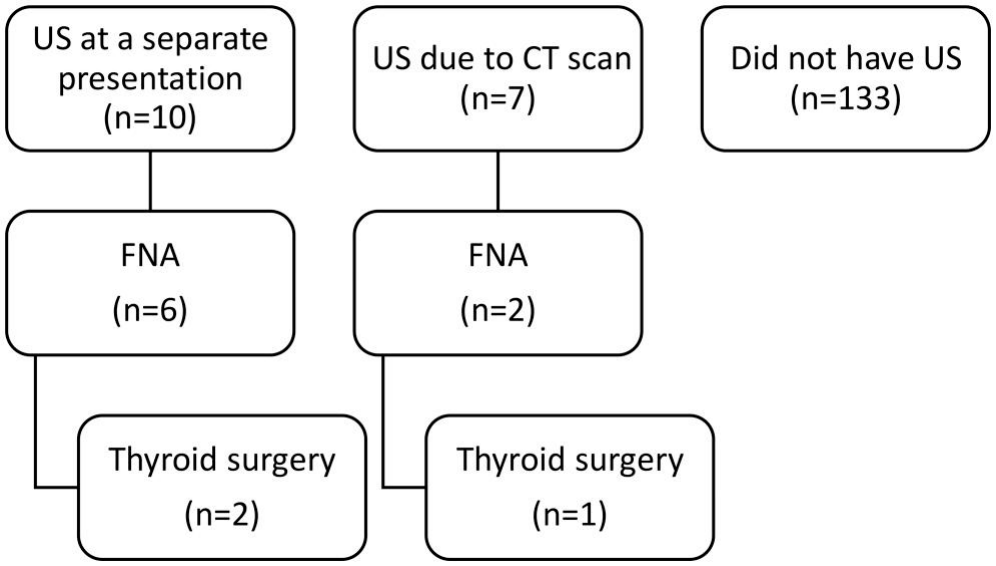
Further investigations and procedures during the follow-up period. FNA = fine needle aspiration; US = ultrasound.

In January 2009, 4.7% of patients (*n* = 7) had thyroid ultrasound due to the CT scan report. Additionally, 6.7% (*n* = 10) of patients had a thyroid ultrasound for a separate presentation in the follow-up period. None of the 17 cases showed suspicious features on ultrasound. 5.3% (*n* = 8) of patients underwent FNA for their thyroid nodule in the follow-up period with no malignant cytology and 2% (*n* = 3) patients underwent hemithyroidectomy, all benign nodules histologically. Figure 6 summarizes the further investigations and procedures.

### Further cross-sectional imaging

Fifty-six percent (*n* = 84) of patients had further cross-sectional imaging that covered the thyroid in the follow-up period. The mean time to the second scan was 1654 (±1432) days, approximately 4.5 years. In 70% (*n* = 59), nodule size did not change. In 14% (*n* = 12), the nodule had increased in size. In 6% (*n* = 5), the nodule had decreased in size. In 9.5% (*n* = 8), the nodule had disappeared. In the 12 nodules which increased in size, the mean increase was 7.0 mm (±4.4), with maximum growth rate of 1.88. In the 5 nodules, which decreased in size during the follow-up period, the mean decrease was 6.3 mm (±2.0).

## Discussion

We found the prevalence of ITN ≥10 mm on a series of consecutive CT scans, based on direct assessment of the images, to be 11%. Of 150 patients with ITN, none presented with a clinically evident thyroid cancer over a 13-year follow-up period, with 80% of patients deceased by the study endpoint from unrelated causes.

Just under a third of these ITNs were reported on the formal CT report with recommendation for further investigation in just under a quarter. Of the 84 patients who had a further CT scan at a mean interval of 4.5 years, the majority of ITNs were unchanged, reduced in size or no longer present with a minority showing a modest increase in size.

Our study prevalence of 11% is within the range found in previous studies although the wide range reflects differences as to how ITN prevalence is determined. Park et al.^10^ found a prevalence of ITNs on contrast enhanced thoracic CT scans during a 10-year period of 4.5% while Drake et al.^11^ found a rate of 5.8% and 5.1% in contrast enhanced and unenhanced CT scans of the chest, respectively. In both of these studies, the prevalence was determined from searching key words in the radiology report. On the other hand, Yoon et al.^12^ found ITN prevalence of 16.8% and Ahmed et al.^13^ a rate of 25% on dedicated review of images from contrast enhanced thoracic CT. In both of these latter studies, however, the majority of ITNs were less than 10 mm, unlike in our study where only nodules ≥10 mm were included as this was felt to be more relevant to clinical practice and in line with the ACR size threshold for recommending further investigation, at least in younger patients. In addition, we included all types of CT scan that included the thyroid and did not discriminate between contrast and unenhanced CT as this was felt more typical of day-to-day practice in a radiology department.

Many studies suggested that female sex is associated with presence of thyroid nodules.^13^^,^^14^ Our results align with this, with approximately two thirds of the ITN cases being female.

In our study sample, the mean age of those with ITN was 70 years, similar to Park et al.^10^ who found mean age of 69. The majority of studies demonstrate an association between increasing age and presence of thyroid nodules.^15^^,^^16^ Ahmed et al.^13^ found that there was a significant difference in mean age between patients with no thyroid nodules and patients with at least 1 thyroid nodule. We did not examine the demographics of the patients who did not have an ITN ≥10 mm.

Perhaps the most striking result is that 80% of patients were deceased at the study end-point, all from non-thyroid related disease. CT scan was performed for cancer staging or respiratory disease in 68% of the patients. This outcome puts into context the importance of considering comorbidities and life expectancy before recommending further investigation of ITN found at cross-sectional imaging and embarking on a cascade of further thyroid imaging ± biopsy and surgery.

The ITN was reported either in the main body and/or conclusion of the CT report in 30%. It is clear that routine clinical reporting of ITN results in prevalence far lower than on dedicated image review.^17^ Reporting practices also vary widely amongst radiologists depending on nodule size and number, patient past medical history, and subspecialist expertise.^18^^,^^19^ Recommendation for further investigation was made in 10 patients (22%). A small proportion of patients, 11 in total, were subsequently seen at clinic regarding their thyroid nodule and 17 patients had subsequent thyroid ultrasound but both clinic attendance and ultrasound were just as likely if their nodule had not been reported on the CT. It is well recognized that there is inconsistency between ITNs reported by radiologists and ITNs that receive further investigation by clinicians.^20^ Our sample included a relatively high proportion of elderly patients with comorbidities, therefore lower rates of further investigation could be reflective of this. Ultimately, 8 patients proceeded to FNA and 3 to hemithyroidectomy with no malignant cytology or histology found.

While most of the ITNs remained static, reduced in size, or had resolved, a minority (14%) increased in size when measured at subsequent CT performed for non-thyroid reasons during the follow-up period. None had doubled in size since the highest growth rate was 1.88. However, doubling time is not an established indicator of malignancy in thyroid nodules.^21^ Several previous studies suggest that there is no significant difference between growth rates of malignant and benign thyroid nodules^22^^,^^23^ while other studies show that malignant thyroid nodules grow faster than benign ones.^24^^,^^25^ Additionally, nodule shrinkage has not been shown to distinguish between benign and malignant nodules.^25^ Overall, there is limited evidence on the association between the growth rate of thyroid nodules and their risk of malignancy.

In agreement with Ahmed et al.^26^, Yoon et al.^12^ concluded that further investigations should be performed for an ITN detected on CT if there are nodule characteristics suggesting malignancy, including calcification. Khoo et al.^27^ suggested that thyroid surgery should be performed regardless of FNA results in patients with calcified solitary thyroid nodules. On the other hand, Hoang et al.^28^ and Nguyen et al.^29^ found no evidence to suggest increase in malignancy rate with calcification in ITNs. In our study sample, presence of calcification in some form was detected in almost a third of cases. Eleven of those were solitary nodules; however, this had no association with development of clinical malignancy. Sixteen out of 48 were reported, with action recommended in 2. This suggests that nodule calcification was not particularly seen as a predictor of malignancy by the majority of reporting radiologists in 2009.

Our findings would support the recommendations from BTA and NICE, that ITNs detected on cross-sectional imaging do not require further investigation if there is no evidence of infiltrative features or clinical concern for thyroid malignancy. Limiting over-investigation of thyroid lesions which would often prove to be benign or indolent in the long term can reduce the risk of harm to patients caused by additional unnecessary procedures,^30^ diminish stress in patients,^31^ and decrease the cost for futile work-up.

With increasing quality and resolution of imaging techniques, it is likely that more ITNs are being reported on unrelated CT in the present day compared to previous years. There is lack of consistent approach to reporting and recommending further investigations for ITNs detected on CT scans, highlighting the need for a more standardized approach.

This retrospective cohort study investigated the clinical and imaging outcomes of ITNs detected on CT scans for the longest follow-up period to our knowledge. One of the strengths of this study is the large sample size with 1499 CT scans being directly examined for presence of ITNs. In addition, there were only few exclusion criteria, which provided a study highly reflective of everyday clinical practice. No patients had clinical, sonographic, cytological, or histological thyroid malignancy found in the follow-up period. This would make it difficult to draw any conclusions regarding association between nodule size and composition and rate of malignancy. However, it is reassuring that had these nodules been left alone, no clinically relevant malignancies would have been missed. Most of the sample population were aged 65 years and above, with comorbidities, mainly non-thyroid malignancy or respiratory disease. Therefore, mortality in these cases was often related to these pre-existing conditions. However, this is reflective of real-world practice.

Limitations of this study include the fact that it is a retrospective cohort study and that determination of whether there was development of thyroid cancer was mostly based on electronic record follow-up. Only a small proportion of patients had biopsy and few had thyroid surgery. Some patients might have been lost to follow-up and some patients’ electronic health records might have been incomplete.

Long-term follow-up of ITNs detected on CT in a different cohort of patients who are of a younger age with no pre-existing comorbidities could provide further useful information. Reporting practices surrounding ITNs could have changed immensely over the past decade. Since our results reflect the practices in 2009, one of the further questions that would be interesting to evaluate is how today’s clinical practice compares to our data in terms of reporting ITNs, offering further recommendations and rate of ultrasound follow-up, biopsy, and surgery.

## Conclusions

In our study, none of the 150 cases of ITNs developed clinically apparent thyroid malignancy in the 13-year follow-up period. All the cases that were further investigated demonstrated no malignant features. 80% were deceased due to other causes by the study endpoint. This study supports the recommendation that ITNs detected on CT scan do not require further investigation unless invasive features are present or there is significant clinical concern for malignancy. Certainly, patient comorbidities should be taken into consideration prior to reporting ITNs and/or recommending further investigation.
